# Expression and Correlation of Cell-Free cIAP-1 and cIAP-2 mRNA in Breast Cancer Patients: A Study from India

**DOI:** 10.1155/2020/3634825

**Published:** 2020-08-25

**Authors:** Amit Kumar Verma, Irfan Ahmad, Prasant Yadav, Arshad Husain Rahmani, Bazila Khan, Mohammed A. Alsahli, Prakash C. Joshi, Hafiz Ahmad, Mirza Masroor Ali Beg

**Affiliations:** ^1^Department of Zoology and Environmental Sciences, GKV, Haridwar, India; ^2^Department of Clinical Laboratory Science, College of Applied Medical Sciences, King Khalid University, Abha, Saudi Arabia; ^3^Research Center for Advanced Materials Science, King Khalid University, Abha, Saudi Arabia; ^4^Department of Biochemistry, All India Institute of Medical Sciences, Bhopal, Madhya Pradesh, India; ^5^Department of Medical Laboratories, College of Applied Medical Sciences, Qassim University, Buraydah, Saudi Arabia; ^6^School of Biotechnology, Gautam Buddha University, Noida, Uttar Pradesh, India; ^7^Department of Medical Microbiology and Immunology, RAK Medical & Health Sciences University, Ras Al Khaimah, UAE; ^8^Department of Biochemistry, Maulana Azad Medical College, New Delhi, India; ^9^Department of Toxicology, Jamia Hamdard, New Delhi, India

## Abstract

**Background:**

Inhibitors of apoptosis proteins such as cIAP-1 and cIAP-2 have recently emerged as the key mechanism in resistance to apoptosis in various cancers and lead to cell survival. Therefore, the present study aimed to evaluate the cIAP-1 and cIAP-2 expression in breast cancer patients, as well as their association with overall patient survival.

**Methods:**

Histopathologically confirmed 100 invasive ductal carcinoma patients and healthy controls were included in the present study. Total RNA extraction was done from the serum sample of the patients; further, 100 ng of total RNA was used to synthesise cDNA from patients' as well as from healthy controls' serum. Quantitative real-time PCR was performed using the maxima SYBR Green dye to study the expression of cIAP-1 and cIAP-2, and beta-actin was used as the internal control.

**Results:**

The study observed that breast cancer patients had 13.50 mean fold increased cIAP-1 mRNA and 8.76 mean fold increased cIAP-2 mRNA expression compared to the control subjects. Breast cancer patients in the TNM stages I, II, III, and IV showed 9.54, 11.80, 15.19, and 16.83 mean fold increased cIAP-1 mRNA expression (*p*=0.004). Distant organ metastasis, (*p*=0.008), PR status of breast cancer patients (*p* < 0.0001), and HER2 status of breast cancer patients (*p* < 0.0001) were found to be associated with cIAP-1 mRNA expression. Breast cancer patients with different TNM stages such as stages I, II, III, and IV showed 7.8, 8.09, 7.97, and 12.85 mean fold increased cIAP-2 mRNA expression (*p*=0.0002). Breast cancer patients with distant organ metastases status were found to be associated with cIAP-2 mRNA expression (*p* < 0.0001). Breast cancer patients with <13-fold and >13-fold cIAP-1 mRNA expression showed 37.39 months and 34.70 months of overall median survival, and the difference among them was found to be significant (*p*=0.0001). However, cIAP-2 mRNA expression among <8-fold and >8-fold mRNA expression groups showed 35 months and 27.90 months of overall median survival time (*p* < 0.0001). Higher cIAP-1 mRNA expression was linked with smoking and alcoholism among the breast cancer patients (*p* < 0.0001 and *p* < 0.0001). Significant association of higher cIAP-1 mRNA expression was found with the advancement of the disease, while higher mRNA expression of cIAP-1 was associated with distant organ metastases in ROC curve analysis.

**Conclusion:**

The present study suggested that increased cell-free cIAP-1 and cIAP-2 mRNA expression was correlated with the advancement of disease, progression of disease, and overall reduced patient survival. Cell-free cIAP-1 and cIAP-2 mRNA expression could be the predictive indicator of the disease.

## 1. Introduction

Apoptosis is a highly conserved cell death mechanism carried out by high machinery in our body, and the hallmark step of this mechanism includes the activation of aspartic acid-specific cysteine proteases called caspases [1]. On activation, caspases cleave many substrates within the cell, leading to various structural and physiological alterations associated with apoptotic cells. These apoptotic events include DNA fragmentation, chromatin condensation, nuclear membrane breakdown, and formation of an apoptotic body which can be phagocytized easily [2]. During apoptosis, permeabilization of the mitochondrial membrane leads to the release of cytochrome-C and SMAC/DIABLO (second mitochondria-derived activator of caspase/direct IAP Binding protein with Low PI) from the mitochondrial intermembranal space into the cytosol [3]. To avoid calamitous consequences of apoptosis, caspase functioning in cells is closely controlled by a family of polypeptides known as IAPs (inhibitors of apoptosis). IAP is the presence of approximately 70 amino acids and at least one and up to three baculovirus IAP repeat (BIR) domains which facilitate its binding with caspases and ultimately lead to caspase inhibition. Among these IAPs (cIAP-1 and cIAP-2) and X-linked IAP (XIAP), there exist structural similarities, such as each have three N-terminal BIR domains, followed by a C-terminal RING finger that has ubiquitin-protein isopeptide ligase activity [4]. Cytoplasmic cIAP-1 and cIAP-2 have been shown to inhibit caspases-3, 7, and 9 by blocking the caspase-active sites with their zinc finger-like BIR domains that directly bind to active caspases [5, 6]. Overexpression of cIAP-1 and cIAP-2 has diminished the effect on apoptotic activity of the cell, leading to unwanted growth and proliferation, a basic feature of carcinomas [7]. Several studies have suggested the role of IAPs in various signal transduction pathways through which they regulate apoptosis. cIAP-1 and cIAP-2 suppress caspase-8 activation and regulate nuclear factor kappa B (NF-*κ*B) signaling in response to the tumor necrosis factor (TNF-*α*) [8]. cIAP-1 and cIAP-2 also play an important role in TNFR signaling by interacting with TRAF1 and TRAF2. It has been seen that cIAP-1 and cIAP-2 can ubiquitylate TRAF2 and TRAF1, respectively, and can mediate them for proteasome-dependent degradation [9]. It also has been reported that IAPs interact with each other to further enhance their stability and anticaspase activity [10]. Salvesen and Duckett [10] expressed IAP family proteins (cIAP-1 and cIAP-2), tested their association with survivin, and found that both cIAP-1 and cIAP-2 are bound to the survivin protein directly to form the IAP-IAP complex that inhibits apoptosis. Various studies have been conducted in the past to establish the role of SMAC in IAP. SMAC tends to promote caspase-9 activation by neutralizing the inhibitory effect on caspases by interacting with cIAP-1 and cIAP-2 [11].

## 2. Materials and Methods

### 2.1. Patient Blood Sample Collection and Total RNA Extraction

A total of 100 histopathologically confirmed breast cancer patients and 100 healthy controls were recruited for the study. Three millilitres (ml) of the peripheral blood sample was collected in plain vials, and the serum was separated. Total RNA extraction was done from the serum using the Trizol (Invitrogen) reagent as per the instruction given by the manufacturer and stored at −80°C until an additional necessary step like cDNA synthesis was done. The quality and purity of RNA were determined by the A260/280 ratio using a nanospectrophotometer. This study was ethically approved by the ethical committee, and the work was conducted at Gurukula Kangri University, Haridwar, India.

### 2.2. Complementary DNA Synthesis and Quantitative Real-Time PCR for cIAP-1 and cIAP-2 mRNA Expression

100 ng of total RNA was used to synthesise cDNA, following manufacturer's protocol (Verso, Thermo Scientific, USA). The cIAP-1 and cIAP-2 mRNA expression was done by quantitative RT-PCR using SYBR Green I technology, and the beta-actin gene was used as the housekeeping control to analyse the fold change in mRNA expression. The primer sequences for cIAP-1 and cIAP-2 mRNA and beta-actin mRNA amplification are depicted in Table 1. The cIAP-1 and cIAP-2 mRNA expression study was executed by using the programme for 40 cycles, with the first denaturation step at 94°C for 35 s, annealing was for 40 s to 600°C with various temperatures, and extension was done at 72°C for 40 s, and the final reaction volume was maintained to 20 *μ*l. The final step for extension was at 72°C for 5 minutes. Melting curve analysis was done between the temperature ranges of 35°C and 90°C for target amplification, and all processes were performed in duplicate to avoid errors. The relative quantification method, 2-(∆∆CT) method, was used to calculate the cIAP-1 and cIAP-2 mRNA expression using beta-actin as the in-house control, and finally, the results were expressed as the mean fold change in breast cancer patients compared to controls.

### 2.3. Statistical Analysis

All the data were analysed by using the SPSS 16 version and Graph Pad version 5.03. On the basis of data observation, parametric and nonparametric statistical methods were used to analyse the association of the outcome with different variables. Fold change in cIAP-1 and cIAP-2 mRNA expression was analysed by the 2-(∆∆CT) method. Kaplan–Meier analysis was done to compute breast cancer patient survival. A *p* value <0.05 was considered as statistically significant.

## 3. Results

### 3.1. Demographic and Clinical Characteristic of Study Subjects

The demographic and clinical characteristics of 100 breast cancer patients and 100 healthy controls are depicted in Table 1. All patients and healthy controls were females and divided into two age groups: <45 years and >45 years. In the breast cancer patient <45 years of age group, the patient percentage was 32%, and in the >45 years of age group, it was 68%, while healthy controls were 30% and 70%, respectively. Further details of the patient characteristics are mentioned in Table 2.

### 3.2. cIAP-1 mRNA Expression and Clinicopathological Feature of Breast Cancer Patients

It was observed that breast cancer patients had 13.50 mean fold increased cIAP-1 mRNA expression compared to the control subjects (Table 3). Breast cancer patients in TNM stages I, II, III, and IV showed 9.54, 11.80, 15.19, and 16.83 mean fold increased cIAP-1 mRNA expression, and the difference among them was found to be statistically significant, respectively (*p*=0.004). Breast cancer patients with distant organ metastases showed 16.83 mean fold increased mRNA expression, while nonmetastatic breast cancer patients showed 12.92 mean fold increased cIAP-1 mRNA expression, and the difference among them was found to be significant (*p*=0.008). Patients with PR- (progesterone receptor-) positive results showed 19.57 mean fold cIAP-1 mRNA expression, while patients negative for PR showed 10.09 mean fold cIAP-1 mRNA expression (*p* < 0.0001). It was observed that patients with HER-2-positive status showed 17.16 mean fold cIAP-1 mRNA expression, while patients with HER-2-negative status showed 9.37 mean fold cIAP-1 mRNA expression (*p* < 0.0001). Higher mRNA expression of cIAP-1 was observed among breast cancer patients who were smokers compared to nonsmoking breast cancer patients (*p* < 0.0001). Patients who were alcoholic also showed a higher mRNA expression of cIAP-1 compared to nonalcoholic patients (*p* < 0.0001).

### 3.3. cIAP-2 mRNA Expression and Clinicopathological Feature of Breast Cancer Patients

In patients, 8.76 mean fold increased cIAP-2 mRNA expression was observed compared to control subjects (Table 4). Breast cancer patients with different TNM stages such as stages I, II, III, and IV showed 7.8, 8.09, 7.97, and 12.85 mean fold increased cIAP-2 mRNA expression, respectively, and the difference among them was found to be significant (*p*=0.0002). Breast cancer patients with distant organ metastases showed 12.85 mean fold increased cIAP-2 mRNA expression, while patients without distant organ metastases showed only 8.04-fold cIAP-2 mRNA expression, and the difference among them was found to be significant (*p* < 0.0001). However, no such differences in cIAP-2 mRNA expression were seen among other variable groups. No impact of smoking and alcoholism was observed on cIAP-2 mRNA expression among breast cancer patients.

### 3.4. Correlation of cIAP-1 and cIAP-2 in Breast Cancer Patients

**Figure 1:**
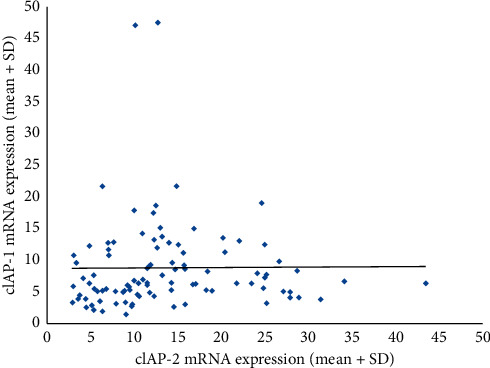
Correlation between cIAP-1 and cIAP-2 among breast cancer patients.

A positive correlation was observed between cIAP-1 and cIAP-2 in breast cancer patients (Figure 1). The observed correlation coefficient (*r*) was 0.21, and the observed *p* value was 0.03. This showed that an increase in cIAP-1 will tend to increase the cIAP-2 and vice versa.

### 3.5. Survival Analysis of Breast Cancer Patients with respect to cIAP-1 and cIAP-2

**Figure 2:**
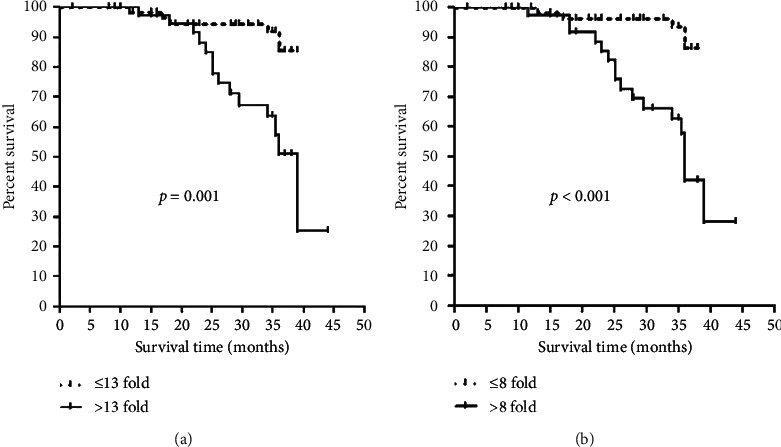
Kaplan–Meier survival curve analysis: (a) cIAP-1 mRNA expression w.r.t breast cancer and (b) cIAP-2 mRNA expression w.r.t breast cancer.

Survival analysis was done to calculate the median survival of breast cancer patients, based on the mean fold expression of cIAP-1 and cIAP-2, and patients were divided into two groups (Figures 2(a) and 2(b)). For cIAP-1 mRNA expression, groups of <13 fold and >13 fold were formed, and it was observed that the median survival of breast cancer patients with <13-fold cIAP-1 mRNA expression showed 37.39 months of overall median survival time, while the >13-fold cIAP-1 mRNA expression group had 34.70 months of overall median survival, and the difference among them was found to be significant (*p*=0.001).

For cIAP-2 mRNA expression, <8 fold and >8 fold groups were formed, and it was observed that the median survival of breast cancer patients with <8-fold cIAP-2 mRNA expression showed 35 months of overall median survival time, while >8-fold cIAP-2 mRNA expression had 27.90 months of overall median survival, and the difference among both groups was found to be statistically significant (*p* < 0.0001).

### 3.6. Prognostic Importance of cIAP-1 and cIAP-2 mRNA Expression for TNM Stages among Breast Cancer Patients

**Figure 3:**
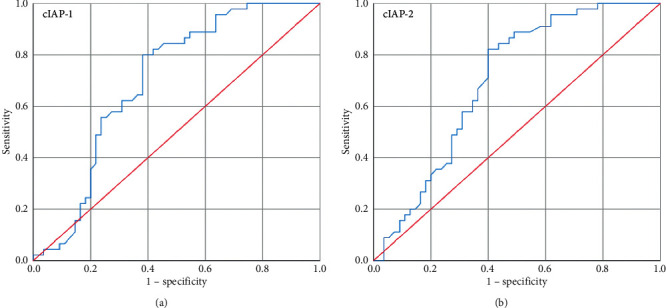
ROC curve (a) for cIAP-1 with respect to early stage vs. advanced stage and (b) for cIAP-2 with respect to early stage vs. advanced stage.

To examine the prognostic significance of cIAP-1 and cIAP-2 in breast cancer patients, stages were categorized into two groups, and ROC curve analysis was made (Figure 3). The ROC curve with respect to the early stage vs. advanced stage of breast cancer patients showed a possible cutoff value of 10.67-fold increase for cIAP-1 and 6.39-fold increase for cIAP-2 and sensitivity was 80% and 71% and specificity was 62% and 60%, respectively (AUC = 0.70, *p*=0.001; AUC = 0.69, *p*=0.001) (Table 5).

### 3.7. Prognostic Importance of cIAP-1 and cIAP-2 mRNA Expression for Distant Organ Metastases among Breast Cancer Patients

**Figure 4:**
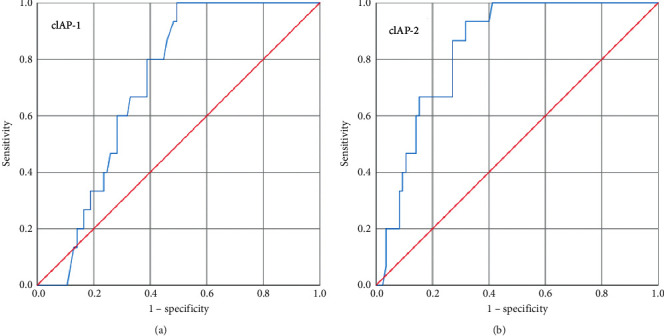
ROC curve (a) for cIAP-1 with respect to no metastases vs. distant organ metastases and (b) for cIAP-2 with respect to no metastases vs. distant organ metastases.

To examine the prognostic significance of cIAP-1 and cIAP-2 in breast cancer patients, two groups were made: no distant organ metastases and distant organ metastases, and the ROC curve analysis was made (Figure 4). The ROC curve with respect to no distant organ metastases vs distant organ metastases of breast cancer patients showed a possible cutoff value of 12.51-fold increase for cIAP-1 and 8.11-fold increase for cIAP-2 and sensitivity was 73% and 86% and specificity was 62% and 70%, respectively (AUC = 0.71, *p*=0.009; AUC = 0.83, *p* < 0.0001) (Table 6).

## 4. Discussion

Apoptosis modulation-linked molecules can be considered as a new approach in combination with other chemotherapeutic drugs for cancer therapy. This includes important signaling molecules such as inhibitor of apoptosis (IAP) family proteins, which inhibit the apoptosis process. Apoptosis is controlled by IAPs via inhibition and modulation of caspases and the nuclear factor NF-*ĸ*B transcription factor [12, 13]. A variety of SMAC mimetics (SM) that can mimic the SMAC and IAP interaction have been developed and showed to accelerate apoptosis by antagonizing IAP action. More specifically, SMs can inhibit XIAP, rapidly induce degradation, and modulate autocrine TNF-*α* production and activity for the apoptosis process [14–17].

In the present study, we found that 13.50 mean fold increased cIAP-1 and 8.76 mean fold increased cIAP-2 mRNA expression was observed in breast cancer patients. Increase in cIAP-1 was observed to be associated with TNM stages of disease, distant metastases, PR, and HER2 status, while cIAP-2 was associated with TNM stages of disease and distant metastases only. Increased mRNA expression of cIAP-1 and -2 was found to be linked with disease aggressiveness and development. It was found that increased cIAP-1 and cIAP-2 mRNA expression was associated with reduced breast cancer patient overall survival. Patients who had <13 mean fold cIAP-1 expression had better survival compared to >13 mean fold cIAP-1 mRNA expression, while <8 mean fold cIAP-2 mRNA expression showed better survival compared to >8 mean fold increased cIAP-2 mRNA expression, suggesting that overall the increased cIAP-1 and cIAP-2 mRNA expression was linked with bad prognosis and poor survival. IAPs constitute a family of proteins which stop cell death and control several important signaling pathways [18]. IAPs are often deregulated in tumors and have been connected with poor prognosis by increasing cancer aggressiveness and therapy resistance [19]. SMAC mimetics (SMs) were used to target cellular cIAP-1, cIAP-2, and XIAP [17, 20], and it was found that compounds augment the cytotoxic activity with traditional chemotherapy drugs and prevent IAP-mediated start of several signaling cascades [21]. It has been revealed that IAP alterations are involved in chemotherapeutic drug resistance or other apoptotic molecules in tumor cells [22]. Overexpression of cIAP-2 appears to be liable for sustained neutrophilia in some cases of chronic neutrophilic leukaemia [23]. cIAP-2 has been actively implicated in induction of TNF to the ubiquitous transcription factor, NF-*ĸ*B, and protection from apoptosis [24].

It has been revealed that the epithelial to mesenchymal (EMT) regulation is possibly controlled by the IAPs as one of the important functions [25]. However, the function of IAPs related to EMT regulation and cancer metastasis is still under investigation. A study by Liu Ji in 2012 highlighted that cIAP-2 acts as a regulatory factor and could be positive regulators of EMT [26]. It has been reported that cIAP-1 and cIAP-2 play an essential role in NF-*ĸ*B signal transduction via K63-linked ubiquitination of RIP1 [27]. Furthermore, cIAP-1 and cIAP-2 are also targets of NF-*ĸ*B-transactivation, suggesting their positive involvement in the regulation of cIAP-1 and cIAP-2 expression [28]. It has been reported that cIAP-1 and cIAP-2 bind to the mitochondrial protein SMAC that competes with caspases for binding to IAPs when released into the cytosol [29]. cIAPs are involved in ubiquitination of several factors such as the TNF receptor-associated factor 2, serine/threonine kinase NIK, receptor-interacting protein 1, and IAP antagonist SMAC [30, 31]. ROC curve analysis showed that at 10.67 fold or greater than 10.67 fold, cIAP-1 mRNA expression could be indicative for the advancement of the disease, while cIAP-2 at 6.39 fold or greater than 6.39 fold could be indicative for the advancement of the disease. For distant organ metastases, a cutoff value of 12.51-fold or greater than 12.51-fold cIAP-1 mRNA expression could be indicative of the distant organ metastases, while 8.11-fold or greater than 8.11-fold cIAP-2 mRNA expression could be indicative for the distant organ metastases.

## 5. Conclusion

The present study suggests that increased expression of cell-free cIAP-1 and cIAP-2 was observed in breast cancer patients mainly with the TNM stage and distant organ metastases. Expression of cIAP-1 and cIAP-2 mRNA in our patient cohort was associated with advanced disease progression, poor disease outcome, and overall decreased patient survival for breast cancer. cIAP-1 mRNA higher expression could be the predictive marker for the advancement of disease, while cIAP-2 could be the indicator for distant organ metastases.

## Figures and Tables

**Table 1:** Primer sequences for the amplification of cIAP1, cIAP2, and beta-actin.

cIAP-1	Annealing temperature (°C)
Forward: 5′-TCAGAATTGGCAAGAGCTGGT R	60°C
Reverse: 5′-AAATGCCTCCGGTGTTCTGA
cIAP-2
Forward:5′- GCTTGCAAGTGCGGGTTTTT R
Reverse: 5′- ACCTTGGAAACCACTTGGCA
Beta-actin
Forward: 5′-CGACAACGGCTCCGGCATGTGC-3,
Reverse: 5- GTCACCGGAGTCCATCACGATGC-3'.

**Table 2:** Demographic characteristics of breast cancer patients.

Variables	Breast cancer patients *N* = 100 (100%)	Healthy controls *N* = 100 (*n* = 100%)
Age		
<45	32 (32)	30 (30)
>45	68 (68)	70 (70)
TNM stage		
Stage I	3 (3)	
Stage II	52 (52)	
Stage III	30 (30)	
Stage IV	15 (15)	
Metastases		
Yes	15 (15)	
No	85 (85)	
Lymph node involvement		
Yes	54 (54)	
No	46 (46)	
Menopause		
Yes	62 (62)	
No	38 (38)	
ER		
Yes	41 (41)	
No	59 (59)	
PR		
Yes	36 (36)	
No	64 (64)	
HER2		
Yes	53 (53)	
No	47 (47)	
Smoking		
Yes	28 (28)	
No	72 (72)	
Alcoholism		
Yes	32 (32)	
No	68 (68)	

**Table 3:** Association of clinicopathological features with cIAP-1 mRNA expression in breast cancer patients.

Variables		cIAP-1 mRNA expression	
		Mean ± SD	*p* value
Overall expression		13.50 ± 8.07	—
Age		
<45		13.31 ± 8.69	0.66
>45		13.59 ± 7.83
TNM stage		
Stage I		9.54 ± 2.91	0.004
Stage II		11.80 ± 8.50
Stage III		15.19 ± 8.16
Stage IV		16.83 ± 5.17
Distant metastases		
Yes		16.83 ± 5.17	0.008
No		12.92 ± 8.37
Lymph node involvement		
Yes		13.70 ± 8.72	0.98
No		13.27 ± 733
Menopause		
Yes		13.24 ± 7.24	0.94
No		13.93 ± 8.81
ER expression		
Yes		14.83 ± 9.18	0.28
No		12.58 ± 7.14
PR expression		
Yes		19.57 ± 8.39	<0.0001
No		10.09 ± 5.52
HER2 expression		
Yes		17.16 ± 7.80	<0.0001
No		9.37 ± 6.22
Smoking		
Yes		21.50 ± 7.58	<0.0001
No		10.39 ± 5.83
Alcoholism		
Yes		23.14 ± 6.33	<0.0001
No		8.96 ± 3.54

**Table 4:** Association of clinicopathological features with cIAP-2 mRNA expression in breast cancer patients.

Variables	cIAP-2 mRNA expression	
	Mean ± SD	*p* value
Overall expression	8.76 ± 7.16	—
Age		
<45	9.43 ± 8.85	0.44
>45	10.97 ± 9.98	
TNM stage		
Stage I	7.80 ± 6.42	0.0002
Stage II	8.09 ± 8.00	
Stage III	7.97 ± 3.94	
Stage IV	12.85 ± 4.42	
Distant metastases		
Yes	12.85 ± 4.42	<0.0001
No	8.04 ± 7.33	
Lymph node involvement		
Yes	9.37 ± 8.18	0.83
No	8.80 ± 4.44	
Menopause		
Yes	9.46 ± 8.47	0.52
No	7.62 ± 4.09	
ER expression		
Yes	9.03 ± 7.59	0.73
No	8.89 ± 7.33	
PR expression		
Yes	8.85 ± 7.94	0.87
No	8.07 ± 6.73	
HER2 expression		
Yes	9.30 ± 7.12	0.12
No	8.15 ± 7.24	
Smoking		
Yes	8.96 ± 8.09	0.43
No	8.24 ± 3.93	
Alcoholism		
Yes	9.08 ± 8.30	0.54
No	8.07 ± 3.77	

**Table 5:** AUC curve for cIAP-1 and cIAP-2 with respect to the TNM early stage (I and II) and advanced stage (III and IV) of the disease.

Gene	AUC	Cutoff	Sensitivity (%)	Specificity (%)	*p* value
cIAP-1	0.70	10.67 fold	80	62	0.001
cIAP-2	0.69	6.39 fold	71	60	0.001

**Table 6:** AUC curve for cIAP-1 and cIAP-2 mRNA expression with respect to no metastases and distant organ metastases.

Gene	AUC	Cutoff	Sensitivity (%)	Specificity (%)	*p* value
cIAP-1	0.71	12.51 fold	73	62	0.009
cIAP-2	0.83	8.11 fold	86	70	<0.0001

## Data Availability

The datasets used and/or analysed during the present study are available from the corresponding author.
